# Obstructive sleep apnea in children: prevalence and association with overweight and obesity

**DOI:** 10.3389/frsle.2025.1691091

**Published:** 2026-01-28

**Authors:** Ingibjorg Ingolfsdottir, Laufey Hrolfsdottir, Groa Bjork Johannesdottir, Thorhallur Ingi Halldorsson, Solveig Magnusdottir, Erla Guðbjorg Hallgrimsdottir, Magnus Birkisson, Hannes Petersen

**Affiliations:** 1 Akureyri Hospital, Akureyri, Iceland; 2 Faculty of Medicine, University of Iceland, Reykjavik, Iceland; 3 Institution of Health Science Research, University of Akureyri and Akureyri Hospital, Akureyri, Iceland; 4 Faculty of Food Science and Nutrition, University of Iceland, Reykjavik, Iceland; 5 MyCardio LLC, Denver, CO, United States; 6 Aarhus University, Aarhus, Denmark

**Keywords:** home sleep testing, obesity, overweight, pediatric obstructive sleep apnea, pediatric sleep questionnaire

## Abstract

**Introduction:**

Obstructive sleep apnea (OSA) is an underdiagnosed health condition and in young children, if untreated, may have negative effects on mental and physical health. Although obesity is recognized as a major risk factor for OSA in adults, the association between weight and OSA has not been as well documented in children. This study was conducted to evaluate prevalence of OSA in young children and if there is an association with weight gain, overweight and/or obesity.

**Methods:**

Cross-sectional study, conducted over period of one-year included 29% (*n* = 371) of young children (4–9-years old) living within the general population in the recruitment area. Sleep was evaluated subjectively with the pediatric-sleep-questionnaire (PSQ) and objectively using a home sleep test. Two-nights of >4-h of sleep-duration was required for diagnosis of OSA. Overweight and obesity was evaluated using BMI *z-*score. Multivariable log-binomial regression analysis was used to assess the relationship between OSA and overweight/obesity. The analyses were adjusted for age, sex, history of asthma/allergies and prior adenotonsillar surgery.

**Results:**

Undiagnosed OSA is prevalent in young children or 22.7%; with prevalence of moderate-OSA 16.2% and severe-OSA 6.5%. Each one-unit increase in BMI *z-*score was associated with 1.35-times (CI_95%_:1.22, 1.50) higher risk of having moderate/severe OSA. Childhood overweight (RR = 2.71; CI_95%_1.76, 4.16) and obesity (RR = 2.80; CI_95%_1.75, 4.49) were associated with almost three times the increase higher risk of having moderate/severe OSA. When analyzing BMI *z-*score from 18 months of age to current age (ΔBMI *z-*score), each one-unit increase in BMI *z-*score was associated with 1.24-times (CI_95%_: 1.08, 1.41) higher risk of having moderate/severe OSA.

**Conclusions:**

The prevalence of OSA in young children is higher than previously reported, with weight gain, overweight, and obesity strongly associated with OSA diagnoses.

## Introduction

Sleep plays an important role in mental and physical health. Obstructive sleep apnea (OSA) is the most common sleep disorder in children and, if left untreated, may lead to significant short- and long-term complications affecting cardiovascular-, cardiometabolic-, and neurocognitive health. These complications may, in some cases, be irreversible (Beebe, 2006; Smith and Amin, 2019; Song et al., 2016). OSA is characterized by prolonged complete or partial upper airway obstruction, which can lead to intermittent hypoxemia and sleep fragmentation. Night-time symptoms include snoring, gasping, apneas, unusual sleeping positions and nocturnal enuresis, daytime symptoms include morning headaches, sleepiness, inattention, and hyperactivity (Berry et al., 2012). Previous studies have estimated the prevalence of OSA in children to be up to 5.7% (Marcus et al., 2012b) but a recent systematic review suggests higher rates among young children (12.8–20.4%) with prevalence increasing over the past decade (Magnusdottir and Hill, 2024).

The most common risk factors for OSA in young children include tonsillar and/or adenoid hypertrophy. Early adenotonsillectomy in children with OSA has been shown to reduce all-cause health care use (Bakker et al., 2025). However, adenotonsillectomy is less likely to resolve OSA in children who are overweight or have obesity compared to healthy weight children, suggesting obesity plays a role in the pathophysiology of OSA (Bhattacharjee et al., 2010; Imanguli and Ulualp, 2016). Prevalence of pediatric obesity has increased worldwide over the past five decades (1975-2016) with the global age-standardized prevalence of obesity in children and adolescents aged 5–19 years increasing from 0.7% to 5.6% in girls and from 0.9% to 7.8% in boys (Abarca-Gómez et al., 2017). This global epidemic of pediatric obesity is likely to have contributed to the increase in prevalence of pediatric OSA. Although obesity is recognized as a major risk factor for developing OSA in adults, the association between weight and OSA is less well documented in children than adults (Keefe et al., 2019; Senaratna et al., 2017).

Despite increased knowledge of the importance of good and regular sleep for physical and mental health in young children, research utilizing objective sleep evaluation is limited, and available data is largely based on implementing subjective questionnaires. Moreover, most previous studies have focused on evaluating children referred to doctors on suspicion of suffering from OSA or children undergoing obesity treatment, i.e. children who may have increased prevalence of OSA, rather than evaluating population-based cohorts (Magnusdottir and Hill, 2024). The reason for the lack of objective sleep measures when evaluating sleep and OSA in children is likely that the current "gold standard” for objectively evaluating sleep in children, Polysomnography (PSG), requires both specialized skillsets to perform and interpret the results, making the test both costly and not widely available (Berry et al., 2012). However, there has been growing demand for access to diagnostic modalities for pediatric OSA as is currently accessible for adults, with increase in research on the validation and utilization of home sleep testing in pediatric populations. Recent studies have demonstrated that new and more convenient sleep testing devices like level III home sleep apnea tests (HSAT) can be used to detect moderate to severe cases of OSA in children (Landry et al., 2024; Teplitzky et al., 2023).

Cardiopulmonary-coupling (CPC), a method based on analysis of marked peripheral arterial tone, and autonomic activity in respiratory and cardiovascular interaction, provides an integrated output of electrocortical modulation of cardiovascular and respiration-autonomic interactions during sleep. An embodiment of this technology is the SleepImage^®^ System (Food and Drug Administration, FDA-cleared and European Union Medical Device Regulatory EU-MDR CE-marked compliant). The input signals for the analysis are pulse rate variability (PRV) and respiratory tidal volume variability (TVV), and the output sleep stability measures and the Sleep Quality Index (SQI), heavily weighted by stable NREM sleep, or high frequency coupling (HFC). Combining the CPC-output with oxygenation information (SpO_2_), has been validated against PSG with good correlation in a similar population of children. The method has proven to be accurate to calculate an apnea hypopnea index (AHI) with high accuracy, sensitivity and specificity that is MDR-CE and FDA -cleared to diagnose sleep apnea in children from the age of 2-years (Hilmisson et al., 2020).

The aim of this study was to: (1) Evaluate the prevalence of OSA in a population-based sample of young children, 4–9-years of age and (2) Examine the association of weight gain in childhood, overweight/obesity and OSA.

## Methods

### Study design

A community-based cross-sectional study using objective and subjective measures to evaluate prevalence of OSA in 4–9-year-old children (born 2014-2018) residing in Akureyri, Iceland and the surrounding areas. After approval by the National Bioethics Committee (VSN-22-096), the study was registered at the National Library of Medicine (NCT05479201). In collaboration with the Department of Education and Public Health of Akureyri, e-mails introducing the study were sent to parents/guardians of children through kindergartens, primary schools, and nurses at the local health center. Additionally, advertisements were posted in public buildings in Akureyri and on social media. Parents expressing interest in their child/children to participate in the study were invited to a presentation at Akureyri Hospital where the objectives of the study and procedures were introduced and written informed consent was obtained from parents or legal guardian. Children/parents were not financially compensated for their participation in the study. Study methods and results are reported following the Strengthening the Reporting of Observational Studies in Epidemiology (STROBE) statement for cross-sectional studies (von Elm et al., 2007).

### Participants

Data was collected from July 27th 2022, through June 19th 2023, with total of 386 children enrolled in the study (30% of the 1290 children within the defined age group living in the recruitment area)(Statistics Iceland, 2024). Two nights of ≥4 h of sleep duration were required for diagnosis of OSA. Nine children withdrew and four had technically inadequate recordings (i.e., they did not provide two nights with ≥4 h of continuous data). Data from children with severe asthma or other severe respiratory and pulmonary diseases, genetic disorders that may affect neurocognitive development and/or behavior, muscular dystrophy, and other neuromuscular disorders caused data from two children to be excluded from the final analyses, both diagnosed with genetic disorders. Children with more than 25% missing items on the Pediatric Sleep Questionnaire (PSQ) were excluded from that analysis (*n* = 10). As a result, the final dataset for OSA analyses consisted of 371 children (29% of the 1290 children living in the recruitment area) and 361 for PSQ analyses.

### Clinical assessment

Age and sex of participants were registered, and physical measurements were taken with the child dressed in light clothing without shoes, using Seca weight- and stadiometer-scale. Weight and height were registered using one decimal point and body mass index (BMI) *z-*score for age and sex calculated using the software platform used to store health data in Iceland (“Saga”) (Karlberg et al., 2001). Overweight was defined if BMI *z-*score was >1.5 to 2.5 standard deviations (SD) above average BMI *z-*score and obesity when BMI *z-*score was >2.5 SD above average BMI *z-*score. This is in accordance with the criteria from the World Health Organization (“WHO”) (World Health Organization, 2024) and the Children's Hospital in Iceland (Landspitali, n.d.). The children's BMI *z-*score at 18 months of age was accessed through the Saga-platform.

Parents/caretakers were asked to electronically fill out questionnaires about the child's health including questions about allergy, asthma, nutrition, exercise, prior adenotonsillar surgery, medications, history of respiratory infections in the past 12 months, cardiovascular and central nervous system problems, genetic disorders, metabolic and autoimmune diseases, sleep habits, and behavior. In addition, parents were asked to evaluate the child's general health and quality of life.

### Subjective sleep evaluation

The PSQ is a validated, self-administered and standardized questionnaire utilized to subjectively assess children's sleep problems and symptoms of OSA with reported sensitivity of 81%, specificity of 87% and internal consistency (Cronbach's alpha) 0.66-0.89 (Chervin et al., 2000). PSQ evaluates 22 symptoms, i.e., snoring, apneas and breathing difficulties, daytime sleepiness, inattentive and/or hyperactive behavior, and other symptoms that may correlate with pediatric OSA (Chervin et al., 2007). The total score is calculated by dividing the total score, the number of positive answers (ranging from 0–22) by the total number of questions answered (22) and ranges from 0–1. A score of 0.33 or higher is indicative of OSA (Chervin et al., 2000, 2007).

### Objective sleep evaluation

A home sleep test (SleepImage^®^ System; MyCardio LLC) that complies with the EU Medical Device Regulation (MDR CE-mark) and is FDA-cleared (K182618) for children 2-years and older, was utilized to objectively evaluate OSA. The ability of the SleepImage System for diagnosis of OSA has been validated against PSG in a study including 805 children, presenting high sensitivity (0.88–0.95) and specificity (0.84–0.97), depending on OSA severity (Hilmisson et al., 2020). The SleepImage System includes the SleepImage Ring (SR) that collects plethysmography-signal (PLETH), oxygen saturation (SpO_2_) and movement. Data is transferred via Bluetooth to the SleepImage mobile application and uploaded to the SleepImage System for analysis at the end of the sleep-recording for analysis. The sleep analysis is based on cardiopulmonary coupling (CPC), calculating the coupling and coherence between respiratory excursions (TVV) and pulse-rate variability (PRV) derived from the PLETH signal to derive sleep metrics. Non-rapid eye-movement (NREM) sleep is presented as bimodal: (1) stable NREM-sleep (high-frequency coupling, HFC;0.1–0.5HZ) includes all electroencephalogram (EEG) estimated NREM-stage-3 and the stable part of NREM-stage-2, which is associated with periods of stable breathing, non-cyclic alternating pattern (CAP) EEG, increased delta power and blood pressure dipping, (2) unstable NREM-sleep (low-frequency coupling, LFC;0.01–0.1HZ) includes all NREM-stage-1 and the unstable part of NREM-stage-2 on the EEG, which has the opposite characteristics of stable-NREM sleep; associated with sleep instability characterized by variability in tidal volumes, blood pressure non-dipping and CAP-EEG. Wake and REM sleep are identified by very low-frequency characteristics (vLFC; < 0.01HZ) (Al Ashry et al., 2021; Thomas et al., 2005, 2014). The output includes sleep onset, sleep conclusion, sleep duration, total sleep time, wake after sleep onset, sleep efficiency, sleep quality index (SQI) displayed on a scale of 0–100 and when combined with SpO_2_ information, calculates AHI_3%_ (Hilmisson et al., 2020). The SleepImage output is autogenerated. In this study all sleep reports were manually evaluated and overread/edited for accuracy by a sleep specialist blind to all information about participants other than age and sex. The autogenerated sleep output was adjusted when needed for (1) study start/end time, (2) artifacts were removed and (3) respiratory events edited as appropriate.

Sleep was recorded from Thursday through Monday. For a child to be included in the study, two sleep studies with good signal quality of duration >4 hours of continuous sleep measurement were required. OSA severity was based on the number of apnea hypopnea events per hour of sleep presented in the apnea-hypopnea 3% desaturation index (AHI_3%_). In this study OSA was defined as no-OSA, AHI_3%_ < 2; mild-OSA, AHI_3%_ 2-5; moderate-OSA, AHI_3%_ 5-10 and severe-OSA AHI_3%_ >10). To evaluate prevalence of OSA (moderate/severe-OSA) the categorization was based on the night with the higher AHI value. Sensitivity analyses were also performed using the average of the two recorded nights to assess the robustness of the findings.

### Statistical analysis

Assumptions on the normality of model residuals were checked using histograms and QQ-plots. Differences between means of two or more groups were evaluated using the F-test. Categorical variables were described using frequencies and percentages, and the difference between groups was assessed using the Chi-square test. Statistical significance was accepted at P < 0.05. Multivariable log-binomial regression analysis was used to assess the association between children's weight with OSA and PSQ.

The results were presented as relative risk ratios (RR) with 95% confidence intervals (95%CI). These associations were explored for BMI *z-*score entered as continuous variable as well as presenting the relative risk of OSA for overweight children (1.5 ≤ BMI *z-*score < 2.5), and with obesity (BMI *z-*score ≥2.5) relative to those with healthy weight (BMI *z-*score < 1.5). The corresponding associations were also explored for weight gain (ΔBMI *z-*score), i.e., the difference between BMI *z-*score at the current age (4–9 years) and at 18 months of age In these analyses the following covariates, selected a priori based on their potential influence on weight and OSA (Lumeng and Chervin, 2008; Prasad et al., 2020; Xu et al., 2020; Yang et al., 2024) were adjusted for age, sex (model A); as well as history of asthma/allergies and prior adenotonsillar surgery (fully adjusted model B). Collinearity between covariates was assessed prior to inclusion in the models. Missing data were handled by excluding children with >25% missing PSQ items (*n* = 10); height, weight and OSA data were complete for all participants. All statistical analyses were performed using SAS version 9.2.

## Results

### Population characteristics

The mean age of participating children was 6.0 years; 50.7% were boys, and 5.7% and 9.7% had previous history of asthma and allergy, respectively (Table 1). At 18 months, 98.1% (*n* = 308) of the children had a healthy weight (BMI *z-*score < 1.5). However, at recruitment, 78.7% (*n* = 292) of the children were healthy weight (BMI *z-*score < 1.5); 11.9% (*n* = 44) were overweight (1.5 ≥ BMI *z-*score < 2.5), and 9.4% (*n* = 35) had obesity (BMI *z-*score ≥ 2.5). The children's age, sex and history of asthma/allergy or prior adenotonsillar surgery did not differ significantly among children with adverse sleep outcomes (according to AHI_3%_ and PSQ) compared to children with more favorable sleep outcomes (Table 1).

**Table 1:** Characteristics of participants in relation to the scores on the Apnea-Hypopnea Index (AHI) and Pediatric Sleep Questionnaire (PSQ).

**Variables**	**All^a^**	**AHI < 5^b^**	**AHI ≥5^c^**	***P* value**	**PSQ(-)^d^**	**PSQ(+)^e^**	***P* value**
	**(*n* = 371)**	**(*n* = 287, 77.4%)**	**(*n* = 84, 22.7%)**		**(*n* = 238, 65.9%)**	**(*n* = 123, 34.1%)**	
***Demographic variables***							
Age (mean ± SD)	6.0 ± 1.5	6.0 ± 1.4	5.9 ± 1.6	0.50^f^	6.1 ± 1.5	5.8 ± 1.5	0.06^f^
< 6 years (%)	41.0	40.4	42.9	0.70^g^	38.2	45.5	0.18^g^
>6 years (%)	59.0	59.6	57.1		61.8	54.4	
Male (%)	50.7	51.2	48.8	0.57^g^	47.5	58.5	0.06^g^
Caucasian (%)	97.0	96.5	98.8	0.52^g^	96.6	97.6	0.63^g^
***Clinical variables***							
History of asthma (%)	5.7	5.6	6.0	0.52^g^	4.6	8.1	0.18^g^
History of allergy (%)	9.7	9.8	9.5	0.78^g^	10.1	9.8	0.92^g^
Prior adenotonsillar surgery (%)	21.8	20.2	27.4	0.16 ^g^	19.8	26.8	0.12^g^
***Anthropometric variables***							
Current BMI *z-*score (mean±SD)	0.65 ± 1.29	0.48 ± 1.20	1.24 ± 1.41	**< 0.001** ^**f**^	0.50 ± 1.22	0.93 ± 1.37	**< 0.01** ^**f**^
BMI *z-*score < 1.5 (%)	78.7	84.3	59.5	**< 0.01** ^**g**^	81.9	71.5	0.07^g^
1.5 ≥ BMI *z-*score < 2.5 (%)	11.9	8.7	22.6		10.5	15.4	
BMI *z-*score ≥ 2.5 (%)	9.4	7.0	17.9		7.6	13.0	
BMI *z-*score at 18 months (mean ± SD)	0.72 ± 1.13	−0.82 ± 1.07	−0.38 ± 1.26	**< 0.01** ^**f**^	−0.71 ± 1.11	−0.72 ± 1.14	0.97^f^
BMI *z-*score < 1.5 (%)	98.1	98.8	95.7	0.10^g^	99.0	96.3	0.09^g^
1.5 ≥ BMI *z-*score < 2.5 (%)	1.6	1.2	2.9		0.5	3.7	
BMI *z-*score ≥ 2.5 (%)	0.3	0.0	1.4		0.5	0.0	

AHI, apnea-hypopnea index; BMI, Body Mass index; SRDB-PSQ, Pediatric Sleep Questionnaire. Bold p-values indicate statistical significance (*p* < 0.05).

^a^Values are the mean ± standard deviation and percentage for categorical variables.

^b^No or mild obstructive sleep apnea (AHI < 5).

^c^Moderate or severe obstructive sleep apnea (AHI ≥ 5).

^d^Negative PSQ (< 0.33) – less OSA related symptoms.

^e^Positive PSQ (≥0.33) – more OSA related symptoms.

^f^F-test (Type III) of differences among groups.

^g^Chi-square test of differences among groups.

### Sleep apnea

The recorded prevalence of moderate to severe OSA was 22.7% (*n* = 84) (Table 1), with 16.2% (*n* = 60) of participants diagnosed with moderate OSA (AHI_3%_ 5-10) and 6.5% (*n* = 24) with severe OSA (AHI_3%_≥10). Sensitivity analyses using the mean of two nights yielded comparable results, although with a slightly lower prevalence estimate of 17.5% with moderate-severe OSA (Supplementary Table S1). With respect to PSQ, 34.1% had a positive PSQ (>0.33). The sensitivity and specificity of the PSQ compared to the SleepImage measurements were 33% and 65%, respectively (data not shown).

### Weight and sleep apnea

**Figure 1:**
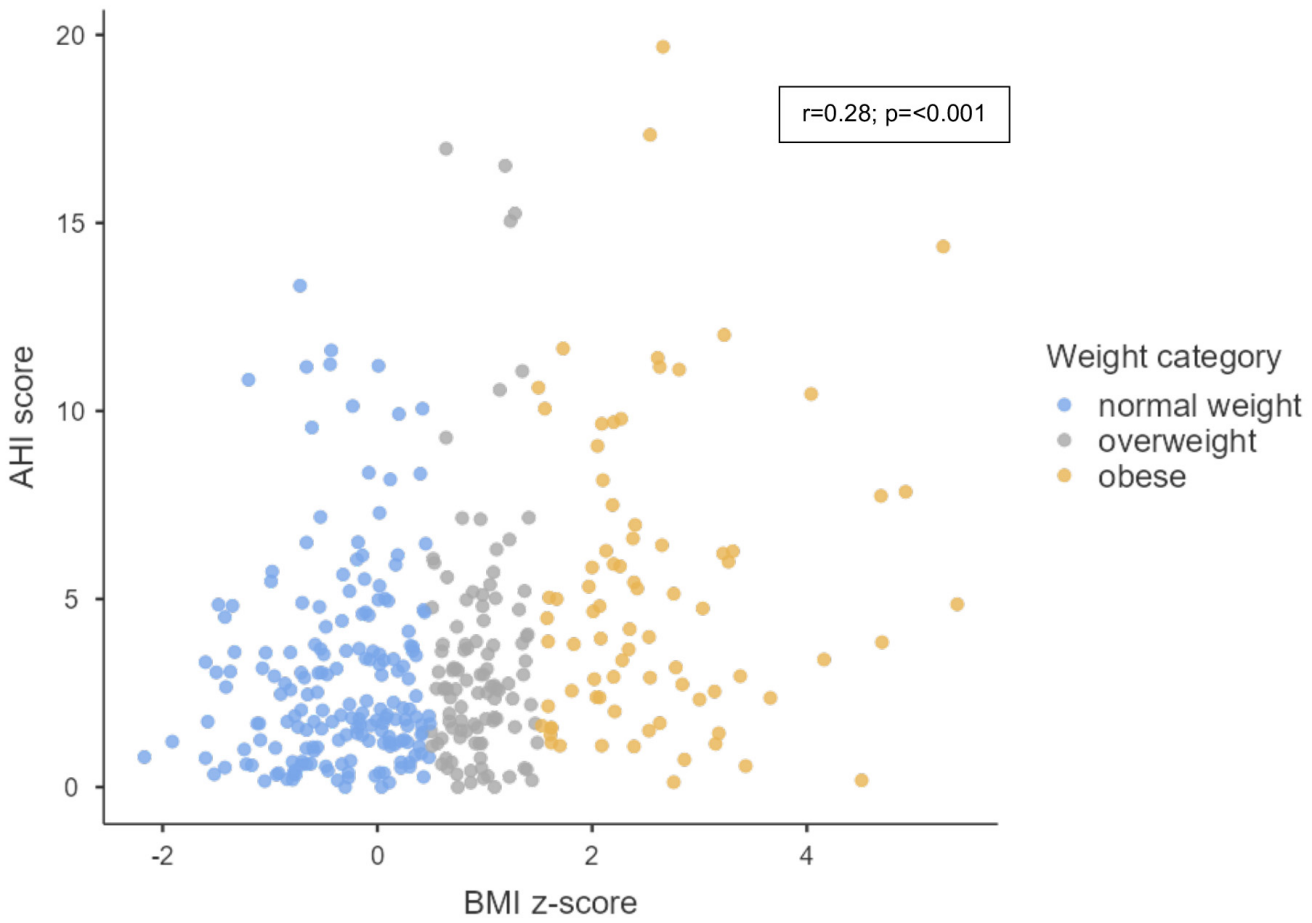
Scatter plot showing the correlation between BMI (Body Max Index) *z-*score and AHI (Apnea Hypopnea Index) score among study participants.

Figure 1 shows the correlation between the BMI *z-*score and the AHI score (*r* = 0.28; *p* = < 0.001). In our fully adjusted model, the association between each 1-unit increase in BMI *z-*score and OSA (Table 2) was 1.35 (95%CI: 1.22, 1.50). The corresponding effect estimates for relative risk of OSA among overweight and obese children relative to those with healthy weight was 2.71 (95%CI: 1.76, 4.16) and 2.80 (95%CI: 1.75, 4.49), respectively. Change in BMI between 18 months and current age was also associated with increased risk of OSA (RR per 1-unit increase in ΔBMI: 1.24 (95%CI: 1.08, 1.41). Sensitivity analyses based on the average AHI across two nights yielded similar results to those obtained when using the higher AHI_3%_ nightly value from the two nights. The associations remained consistent, although slightly attenuated for ΔBMI *z-*score (Supplementary Table S2). As presented in Table 3, significant but slightly weaker associations were observed between children's BMI *z-*score and PSQ.

**Table 2:** The association between BMI *z-*score and moderate to severe OSA (AHI≥5) among 4–9-year-olds.

**BMI *z-*score**		**Unadjusted model**		**Adjusted^b^ model A**		**Adjusted^c^ model B**	
	Cases^*^/n(%)	**RR** ^a^	**95%CI**	**RR** ^a^	**95%CI**	**RR** ^a^	**95% CI**
***BMI categories***							
BMI *z-*score < 1.5	50/292 (17.1%)	Ref	Ref	ref	Ref	Ref	Ref
1.5 ≥ BMI *z-*score < 2.5	19/44 (43.2%)	**2.52**	1.65, 3.84	**2.70**	1.77, 4.11	**2.71**	1.76, 4.16
BMI *z-*score ≥ 2.5	15/35 (42.9%)	**2.50**	1.58, 3.96	**2.77**	1.75, 4.39	**2.80**	1.75, 4.49
*Continuous* ^d^							
BMI *z-*score	84/371	**1.31**	1.20, 1.43	**1.33**	1.21, 1.47	**1.35**	1.22, 1.50
ΔBMI *z-*score^e^	69/314	**1.20**	1.05, 1.37	**1.24**	1.08, 1.43	**1.24**	1.08, 1.41

AHI, Apnea-Hypopnea index; BMI, Body Mass index; RR, Relative Risk; CI, Confidence Interval. Bold values indicate statistically significant results.

^a^Log-binomial regression, reflecting the relative risk of having severe OSA (AHI ≥ 5).

^b^Adjusted for age and sex.

^c^Adjusted for age, sex, history of allergy or asthma and prior adenotonsillar surgery.

^d^Change in RR per 1-unit increase in BMI z-score or change in ΔBMI.

^e^ΔBMI z-score: the difference between BMI z-score at the current age (4–9 years) and at 18 months of age.

^*^Cases of moderate or severe OSA.

**Table 3:** The association between BMI *z-*score and positive Pediatric Sleep Questionnaire (PSQ) among 4–9-year-olds.

**BMI *z-*score**		**Unadjusted model**		**Adjusted^b^ model A**		**Adjusted^c^ model B**	
	Cases^*^/n(%)	**RR** ^a^	**95%CI**	**RR** ^a^	**95%CI**	**RR** ^a^	**95% CI**
***BMI categories***							
BMI *z-*score < 1.5	88/283 (31.1%)	Ref	ref	Ref	ref	Ref	ref
1.5 ≥ BMI *z-*score < 2.5	19/44 (43.2%)	1.39	0.94, 2.03	**1.53**	1.04, 2.23	**1.51**	1.03, 2.21
BMI *z-*score ≥ 2.5	16/34 (47.1%)	**1.51**	1.02, 2.25	**1.48**	1.00, 2.18	1.46	0.98, 2.17
*Continuous* ^d^							
BMI *z-*score	123/361	**1.16**	1.06, 1.27	**1.18**	1.07, 1.29	**1.18**	1.07, 1.30
ΔBMI *z-*score^e^	109/305	**1.17**	1.06, 1.28	**1.16**	1.07, 1.26	**1.16**	1.06, 1.27

AHI, Apnea-Hypopnea index, BMI, Body Mass index, RR, Relative Risk; CI, Confidence Interval. Bold values indicate statistically significant results.

Logistic binomal regression, reflecting the relative risk of a positive pediatric sleep questionnaire score (PSQ ≥ 0.33).

^b^Adjusted for age and sex.

^c^Adjusted for age, sex, history of allergy or asthma and prior adenotonsillar surgery.

^d^Change in RR per 1-unit increase in BMI z-score of change in ΔBMI.

^e^ ΔBMI z-score: the difference between BMI z-score at the current age (4–9 years) and at 18 months of age.

^*^Cases of positive PSQ score.

## Discussion

This community-based cross-sectional study evaluated prevalence of OSA in young children. The study sample is relatively large, both in absolute number and relative to the local population, in comparison with prior studies, including 30% children within the defined age group living in the recruitment area (Statistics Iceland, 2024). The study utilized both a validated home sleep apnea testing device to categorize OSA severity and prevalence based on AHI_3%_ values and subjective questionnaires in all participants. Additionally, association between weight gain in youth, overweight/obesity and OSA was evaluated. The findings are: (1) that prevalence of OSA is higher than previously reported, with 22.7% of the children diagnosed with moderate-severe OSA when diagnosis was based on the more severe night out of the two nights recorded but slightly lower when the nights were averaged, 17.5% and (2) that overweight, obesity and early weight gain are strongly associated with childhood OSA.

Previous studies including a wider age range have estimated prevalence of OSA to be up to 5.7% (Marcus et al., 2012a). A recent systematic review suggests that the prevalence has increased over the past decades (Magnusdottir and Hill, 2024). Results from this community-based cross-sectional study, reporting prevalence of moderate-severe OSA based on AHI_3%_ at 22.7%, align closely with the findings of the systematic review, which reported a prevalence ranging from 12.8% to 20.4% (Magnusdottir and Hill, 2024). A direct comparison with other studies is, however, difficult, as there is a lack of research on OSA epidemiology with currently available data mainly based on subjective questionnaires and lacking objective sleep measurements. Additionally, different methods and definitions have been used to diagnose OSA in children, and very few studies assessing OSA in young children are population based (Magnusdottir and Hill, 2024). In the systematic review of Magnusdottir and Hill (Magnusdottir and Hill, 2024), only seven studies confirmed OSA using objective methods (Ali et al., 1993; Brunetti et al., 2001; Castronovo et al., 2003; Gislason and Benediktsdóttir, 1995; Kaditis et al., 2016; Kitamura et al., 2016; Lofstrand-Tidestrom et al., 1999). However, most of those studies selected participants for objective testing using symptom questionnaires and only implemented objective measures in participants who screened positive for OSA, and not all participants had their sleep objectively measured. One of the most recent study of the seven is from Japan, conducted by Kitamura et al. in 2016; this study reported the highest prevalence of OSA, or 12.8% (AHI≥5) (Kitamura et al., 2016). In this study, parents of 211 children aged 3–6 years were invited to participate, and 89.1% (*n* = 188) completed answering a questionnaire, then 35.6% (*n* = 67) underwent home type-III portable monitoring and clinical examination.

PSQ was utilized for subjective sleep evaluation as it has been validated, is widely used, affordable and easily available (De Luca Canto et al., 2014). Performance of PSQ can differ by age and weight. In this study the age range is narrow but children with adverse sleep outcomes (based on both AHI3% and PSQ) had higher mean BMI *z-*score compared to children with more favorable sleep outcomes. Some studies have suggested PSQ might be ideal in primary care setting as a screening tool in combination with pulse-oximetry and in places where objective sleep tests (HSAT or PSG) are not available while others do not support this type of use for OSA evaluation (Ferry et al., 2020; Wu et al., 2020). The PSQ identified 34.1% of participants as having OSA. The sensitivity and specificity of the PSQ compared to the SleepImage measurements were 33% and 65%, respectively. With the low sensitivity and specificity, PSQ overestimated OSA in children without OSA and underestimated OSA in children with mild, moderate, and severe OSA when compared to the HSAT. These results indicate a weak performance despite the published validation (Chervin et al., 2000, 2007). The validation study was based on children with OSA confirmed by PSG-study in comparison with children visiting a health clinic for any health-related issue that did not undergo PSG-study.

The PSQ is limited by the fact that it is filled out by parents and parental reports may not always accurately reflect the child's actual sleep (Combs et al., 2019). A systematic review of sleep instruments and their validity found that the PSQ lacks sufficient content validity, as it does not comprehensively assess all aspects of sleep health in children (Inhulsen et al., 2022). Furthermore, Caliendo et al., the PSQ does not reliably predict the presence or severity of OSA among children with obesity when compared to objective cardiorespiratory polygraphy (Caliendo et al., 2023).

The weak performance of the PSQ suggests it is not sufficient as a stand-alone diagnostic method for OSA and may not appropriate for use in vulnerable population of young children, underlining the need for objective sleep measures.

Being overweight and having obesity were strongly associated with OSA. At 18 months of age only 1.9% of the children were overweight or had obesity, when at 4–9 years of age 11.9% of the children were overweight, and 9.4% had obesity. Among those that were overweight or had obesity, 40.5% had OSA, which mirrors outcomes from other studies. Alonso-Alvarez et al. (Alonso-Álvarez et al., 2014) estimated that the prevalence of OSA ranged from 21.5% to 39.5% among overweight or obese children ages 3–14 years in the general population in Spain. Similarly, a review by (Verhulst et al. 2008) states that the prevalence of OSA can be as high as 60% among children and adolescents that have obesity. More recent evidence, including a systematic review and meta-analysis, also reported that obesity is associated with an increased risk of OSA among children (2 studies) and adults (10 studies), compared with individuals who did not have obesity (Dong et al., 2020).

The connection between OSA and obesity in the pediatric population is not entirely straightforward, as multiple factors such as muscle tone, fat distribution, inflammation and hormonal regulation interact in complex ways (Bhatt et al., 2021; Zaffanello et al., 2025). Children who are overweight and have obesity may have increased subcutaneous fat in the neck and fatty infiltration of upper airway structures, narrowing the upper airway (Arens et al., 2011). Additionally, accumulation of abdominal fat may increase pressure in the abdominal cavity, causing the diaphragm to have to push against more pressure during breathing (Tauman and Gozal, 2011). The interaction between obesity and OSA is complex, still not fully understood and it is unclear what comes first, OSA or weight gain, raising the possibility of a reciprocal relationship. In this cohort, weight gain from 18 months of age onward was associated with an increased risk of being diagnosed with moderate-severe OSA in young children. However, as sleep data from 18 months were not available, the possibility that OSA itself may contribute to weight gain must also be considered. To better understand this relationship studies evaluating both sleep and weight in young children and tracking them into the adolescence years are required. OSA has been suggested as an independent factor that may affect the metabolic system and thereby contribute to weight gain (Arens and Muzumdar, 2010). In line with this, a longitudinal cohort study among preadolescents (*n* = 319), reported that persistent sleep disordered breathing was associated with an increased risk of developing obesity (Goodwin et al., 2010). Sleep disruption can alter metabolic hormones, including the satiety hormone, leptin and the appetite-stimulating hormone, ghrelin, both of which play important roles in body weight regulation (Cummings and Foster, 2003). Sleep deprivation and sleep fragmentation, are associated with decreased leptin and increased ghrelin levels, which in turn can increase appetite and encourage higher food and energy consumption (Chaput et al., 2007).

Pediatric OSA can have serious long-term physical consequences, such as cardiovascular and metabolic implications, particularly when associated with overweight and obesity, (Thomas et al., 2022), as well as neurocognitive and behavioral impairments (Trosman and Trosman, 2017). Therefore, it is important to identify risk factors early and use valid methods to diagnose and treat children with OSA.

## Conclusion

The results from this population-based study, which implemented objective sleep measures using home sleep apnea testing, suggest that OSA may be an underestimated health problem among young children. The higher prevalence is likely related to the global increase in childhood overweight and obesity, which were strongly associated with OSA in this cohort. Although subjective questionnaires alone are not sufficient to evaluate risk factors for OSA, they can give important information on the manifestation of symptoms among young children. Accessible methods based on validated objective sleep measures are needed to better address sleep disorders in young children.

### Strengths and limitations

The study has several strengths: (1) There are very few population-based studies among children in this age group, especially studies utilizing objective measurements of sleep for the whole cohort in addition to subjective questionnaires; (2) The study sample is relatively large, both in absolute number and relative to the local population, in comparison with prior studies; (3) Low drop-out and failure rate; (4) Prevalence of OSA based on objective sleep measurements for the whole cohort over multiple nights; (5) This is a selective population in a particular area which is a strength because there is less risk for other confounding variables, however, this may limit generalizability overall.

The limitations of the study are: (1) Risk of selection bias, i.e., parents of children with sleep problems or parents who are more interested in their child's/children's sleep may have been more likely to participate. This may introduce a systematic error, and limit the representativeness of the sample, potentially inflating OSA prevalence estimates. With respect to weight status, regional data from the School Health Medical Registry (2022–2023) show that the prevalence of overweight and obesity among school-aged children is 19.5% and 9.1%, respectively, which is comparable to our sample (11.9% and 9.4%) (Development Centre for Primary Healthcare in Iceland (DCPHI), 2023). Although the regional figures span a broader age range, they represent the best available reference for the area and suggest that substantial selection bias related to body weight is unlikely; (2) The analysis is based on the night with the highest AHI_3%_-value, to minimize the risk of missing an OSA diagnosis. This approach may increase the sensitivity of the sleep measurements compared to single-night PSG, which traditional reference ranges rely on. In sensitivity analyses, using the mean of two consecutive nights produced similar results to those obtained from a single night; (3) AHI is the most common method used to diagnose and categorize OSA severity. However, AHI counts the number of apneas/hypopneas per hour of sleep, and does not account for the duration of apneas, and equates apnea and hypopnea as equal events and may therefore poorly reflect the physiological complexities that occur during reduction of airflow, autonomic responses, and sleep fragmentation (Soori et al., 2022); (4) The BMI z score is an accessible parameter for obesity screening in epidemiology and in clinical practice. It is well acknowledged that it is not a perfect measure on the individual level as body composition and fat distribution can differ significantly within the same BMI *z-*score. However, BMI correlates as well as other anthropometric measurements with metabolic syndrome in pediatric obesity (Radetti et al., 2019) and is most relevant for use in primary clinical practice. Guidelines on obesity management commonly advise OSA screening based on BMI percentiles or *z-*scores, though there is a great variability in the precise criteria (Ng et al., 2024);

(5) Information on socioeconomic status, parental education, and family sleep practices was not available in our dataset and could therefore not be included as covariates in the multivariable models. The study population consist of almost only Caucasian Icelandic children and therefore does not reflect ethnic diversity: (6) Though we used a validated device, HSAT's can both under- and over-estimate abnormality. However, by anchoring to a minimum of 3% oxygen desaturations, the likelihood of false positives is reduced. The analysis system also computes total sleep time, which may further reduce errors relative to polysomnography; (7) HSAT cannot currently capture hypoventilation related information.

## Data Availability

The original contributions presented in the study are included in the article/Supplementary material, further inquiries can be directed to the corresponding author.
