# Supplementary material for: Obstructive sleep apnea in children: prevalence and association with overweight and obesity
Source: Front Sleep. 2026 Jan 28;4:1691091. doi: 10.3389/frsle.2025.1691091 (PMC12890620; doi:10.3389/frsle.2025.1691091)
Supplement: Supplementary file 1 [file Data_Sheet_1.pdf]

Obstructive Sleep Apnea in Children: Prevalance and Association with Overweight and Obesity

OBJECTIVE

To evaluate prevalence of moderate and severe Obstructive Sleep Apnea (OSA) in young children and association with weight gain, overweight/obesity

MATERIALS and METHOD

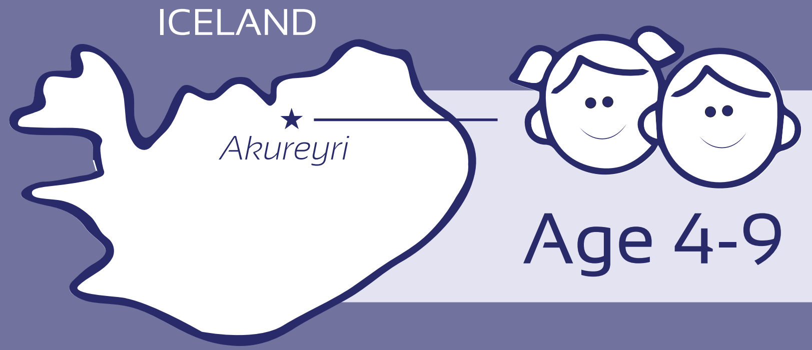

371:
49.3% girls
50.7% boys

- 30% of children living in the study area participated in the study.
- Children were tested for the five nights, 2-nights with >4-hours of sleep required for OSA diagnosis.
- Obesity/ overweight was evaluated with BMI-z-score

FDA-cleared 182618/EU-MDR CE compliant

5 Total Nights

Thu	Fri	Sat	Sun	Mon

(2) Non-School Nights
(3) School Nights

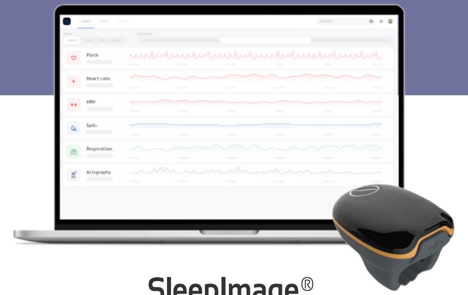

SleepImage®
Home Sleep Testing System

- Sleep Onset
- Sleep Midpoint
- Sleep Conclusion
- Sleep Duration
- Total Sleep Time
- Sleep Efficiency
- Sleep Quality Index
- Apnea-Hypopnea Index
- Sleep Quality Index
- Wake After Sleep Onset

RESULTS

Prevalence of OSA (22.7%) | 16.2% Moderate-OSA | 6.5% Severe-OSA

1PT increase in ⇧ BMI z-score is associated with 1.35 ⇧ higher risk of OSA

Childhood overweight/obesity was associated with 2.8X HIGHER RISK OF OSA

CONCLUSION

Prevalence of OSA in young children is higher than previously reported, with weight gain, overweight, and obesity in childhood being strong predictors of OSA diagnoses.
