# Supplementary material for: Obstructive sleep apnea in children: prevalence and association with overweight and obesity
Source: Front Sleep. 2026 Jan 28;4:1691091. doi: 10.3389/frsle.2025.1691091 (PMC12890620; doi:10.3389/frsle.2025.1691091)
Supplement: Supplementary file 2 [file Table_1.DOCX]

**To Editors of Frontiers in Sleep, "The Promise of Sleep Technology."**

August 22^nd^ 2025

Professor Ambrose A Chiang, MD.

Professor Craig Canapari, MD.

Professor Cathy Goldstein, MD.

Professor Kelly Baron, PHD, MPH, DBSM.

Dear Professors,

On behalf of the authors, I submit this manuscript titled “Prevalence of obstructive sleep apnea in overweight, obese and non-obese children”. We confirm that the manuscript or output related to this study is not under consideration elsewhere and will not be submitted elsewhere while under consideration by Frontiers in Sleep, “*The Promise of Sleep Technology*”.

The existing literature base demonstrates that early intervention to treat Obstructive Sleep Apnea (OSA) in young children improves cognition, cardiovascular health and quality of life and that interventions may possibly be less effective when children reach school age. To understand the possible magnitude of the downstream effects of untreated OSA, knowing the disease prevalence is important. What is the prevalence of OSA in young children? Is the prevalence of a magnitude that might suggest screening for the disease to have the possibility to intervene early with the aim of preventing negative downstream effects of untreated disease and improve health outcomes? What is challenging in pediatric OSA are the dynamic changes that come with the child’s growth and how to consider these changes when determining the appropriateness of therapy and the timing of intervention.

When we initiated this study, the most cited systematic review looking at prevalence of sleep apnea in children was by Lumeng and Chervin published in 2008,^1^ looking at prevalence of OSA in children 2-18 years old from 1950 to 2007, estimating the prevalence 1-4%. The age-range of 2-18 years is wide, including extensive developmental differences with variations in sleep duration, patterns, habits and diseases. Better understanding changes in prevalence of OSA during a more “homogeneous developmental age” might, therefore, be helpful and the age-range of 4-9-years was chosen based on the rapid physical growth taking place, with extensive brain development and changes in sleep patterns happening at the same time when development of adenoids and tonsils may outgrow the cranial bony structures, increasing prevalence of OSA.

During data collection of this submitted manuscript, a systematic review was published in Sleep Medicine Reviews focusing on narrower age group than the Lumeng and Chervin 2008 paper by Magnusdottir and Hill 2024 “*Prevalence of Obstructive Sleep Apnea (OSA) among Preschool Aged Children in the General Population: A Systematic Review*” and includes data from inception through May 2023.^2^ One of the findings of this systematic review was that of the thirty studies included, only seven studies included objective testing and only in a small proportion of participants that screened positive for OSA based on subjective evaluation questionnaires, estimating prevalence of OSA 12.8%-20.4%, with prevalence increasing during the last decade.

The submitted manuscript includes data that was collected from end of July 2022 through mid-June 2023 and includes both objective and subjective evaluation of OSA. The study sample is relatively large, both in absolute number and relative to the local population, in comparison with prior studies, including 371 children in the defined age-range out of a cohort of 1290 children (29%) living in the area. The results of the study demonstrate that the prevalence of OSA is higher than the 1-4% or 22.7%. This study includes additional and updated information that should be relevant for sleep health management in children.

We thank you kindly for your consideration of this submission,

Sincerely,

Ingibjorg Ingolfsdottir

1 ; Lumeng, J.C & Chervin R. D. (2008), Epidemiology of pediatric obstructive sleep apnea. Proc Am Thorac Soc, 5 (2), 242-252.

2: Magnusdottir S & Hill E. (2024). Prevalence of Obstructive Sleep Apnea (OSA) among Preschool Aged Children in the General Population: A Systematic Review. Sleep Med Rev. 2024;73:101871.doi: 10.1016/j.smrv.2023.101871. PMID: 37976758.
